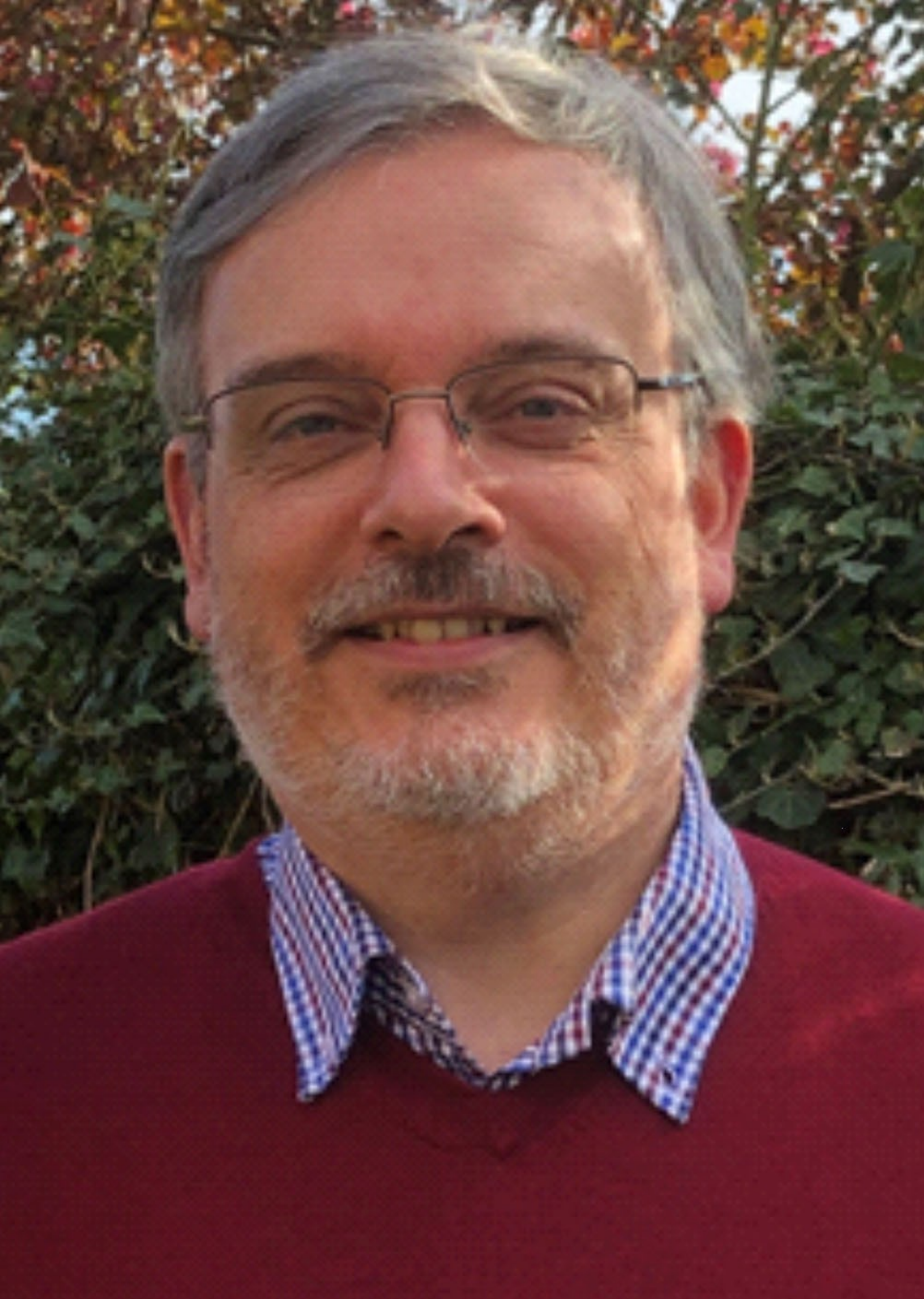# Predictive Engineering of Class I Terpene Synthases Using Experimental and Computational Approaches

**DOI:** 10.1002/cbic.202100484

**Published:** 2021-11-03

**Authors:** Nicole G. H. Leferink, Nigel S. Scrutton

**Affiliations:** ^1^ Future Biomanufacturing Research Hub Manchester Institute of Biotechnology Department of Chemistry School of Natural Sciences The University of Manchester 131 Princess Street Manchester M1 7DN UK

**Keywords:** computational chemistry, functional plasticity, protein engineering, terpene synthases, terpenoids

## Abstract

Terpenoids are a highly diverse group of natural products with considerable industrial interest. Increasingly, engineered microbes are used for the production of terpenoids to replace natural extracts and chemical synthesis. Terpene synthases (TSs) show a high level of functional plasticity and are responsible for the vast structural diversity observed in natural terpenoids. Their relatively inert active sites guide intrinsically reactive linear carbocation intermediates along one of many cyclisation paths via exertion of subtle steric and electrostatic control. Due to the absence of a strong protein interaction with these intermediates, there is a remarkable lack of sequence‐function relationship within the TS family, making product‐outcome predictions from sequences alone challenging. This, in combination with the fact that many TSs produce multiple products from a single substrate hampers the design and use of TSs in the biomanufacturing of terpenoids. This review highlights recent advances in genome mining, computational modelling, high‐throughput screening, and machine‐learning that will allow more predictive engineering of these fascinating enzymes in the near future.

## Graphical abstract

**There is a remarkable** lack of sequence‐function relationship within the terpene synthase family making product‐outcome predictions from sequences alone challenging. Engineering of designer terpene synthases is enabled via integration of multidisciplinary approaches, including generation of sequence diversity, genome mining, high‐throughput product profiling, modelling and computational chemistry, and data‐driven computational tools.

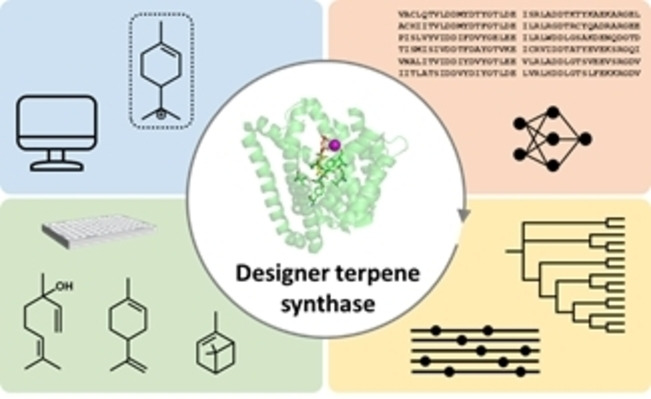

## 1 Introduction

Terpenoids are widely used as flavour and fragrance ingredients, but also as precursors for medicines, bioplastics,^[1]^ and next generation biofuels.^[2]^ The vast majority of these compounds are produced by plants,^[3]^ but bacteria are also increasingly recognised as a rich source of terpenoids.^[4]^ Existing terpenoid production processes have several drawbacks, such as low product titres in native hosts, and stereo‐chemical complexities and use of hazardous solvents for their chemical synthesis. However, recent advances in DNA synthesis and metabolic engineering, have paved the way for the development of engineered microbes for the high‐level production of terpenoids using synthetic biology.^[5]^

Terpenoid biosynthesis is highly modular and all terpenoids are derived from the C_5_ isoprenoid building blocks isopentenyl diphosphate (IPP) and dimethylallyl diphosphate (DMAPP). DMAPP and IPP are produced from acetyl‐coenzyme A via the mevalonate (MVA) pathway in eukaryotes, or from pyruvate and glyceraldehyde‐3‐phosphate via the methylerythritol (MEP) pathway in most bacteria and plant plastids. Alternative pathways, including additional entry points into the MVA and MEP pathways, archaeal MVA pathways, and artificial pathways have also been identified at the sequence level.^[6]^ Prenyl pyrophosphate (prenyl‐PP) synthases catalyse the head‐to‐tail condensation of IPP and DMAPP into prenyl‐PP substrates of varying lengths such as geranyl pyrophosphate (GPP, C_10_), farnesyl pyrophosphate (FPP, C_15_), geranylgeranyl pyrophosphate (GGPP, C_20_) and geranyl‐farnesyl pyrophosphate (GFPP, C_25_), the pre‐cursors for mono‐, sesqui‐, di‐ and sesterterpenoids, respectively. These prenyl‐PP substrates are converted into structurally diverse terpene scaffolds by terpene synthases (TSs).^[7]^

Many examples exist of microbial production of terpenoids using engineered yeast or bacteria, including drug precursors,^[8]^ a range of flavour and fragrance molecules such as limonene and linalool^[9]^ and biofuels.^[10]^ The general strategy involves the introduction of a heterologous pathway for the delivery of the IPP and DMAPP precursors, a prenyl‐PP synthase, and a TS. There are two main bottlenecks associated with this approach.^[11]^ The first is that product titres are generally low, preventing economic production^[12]^ attributed to toxic effects of pathway intermediates and terpenoids which have adverse effects on microbial growth.^[13]^ The second is the broad range of terpenes produced as a result of the inherent functional plasticity of TSs. Efforts to improve titres via optimisation of translational control^[14]^ or fermentation conditions,^[15]^ reduction of toxicity via product modification^[16]^ or the use of cell‐free systems,^[17]^ as well as the use of alternative precursor supply pathways[^10a^, ^18^] have to some extent begun to address these issues but are not the primary focus of this review.

**Figure 1:**
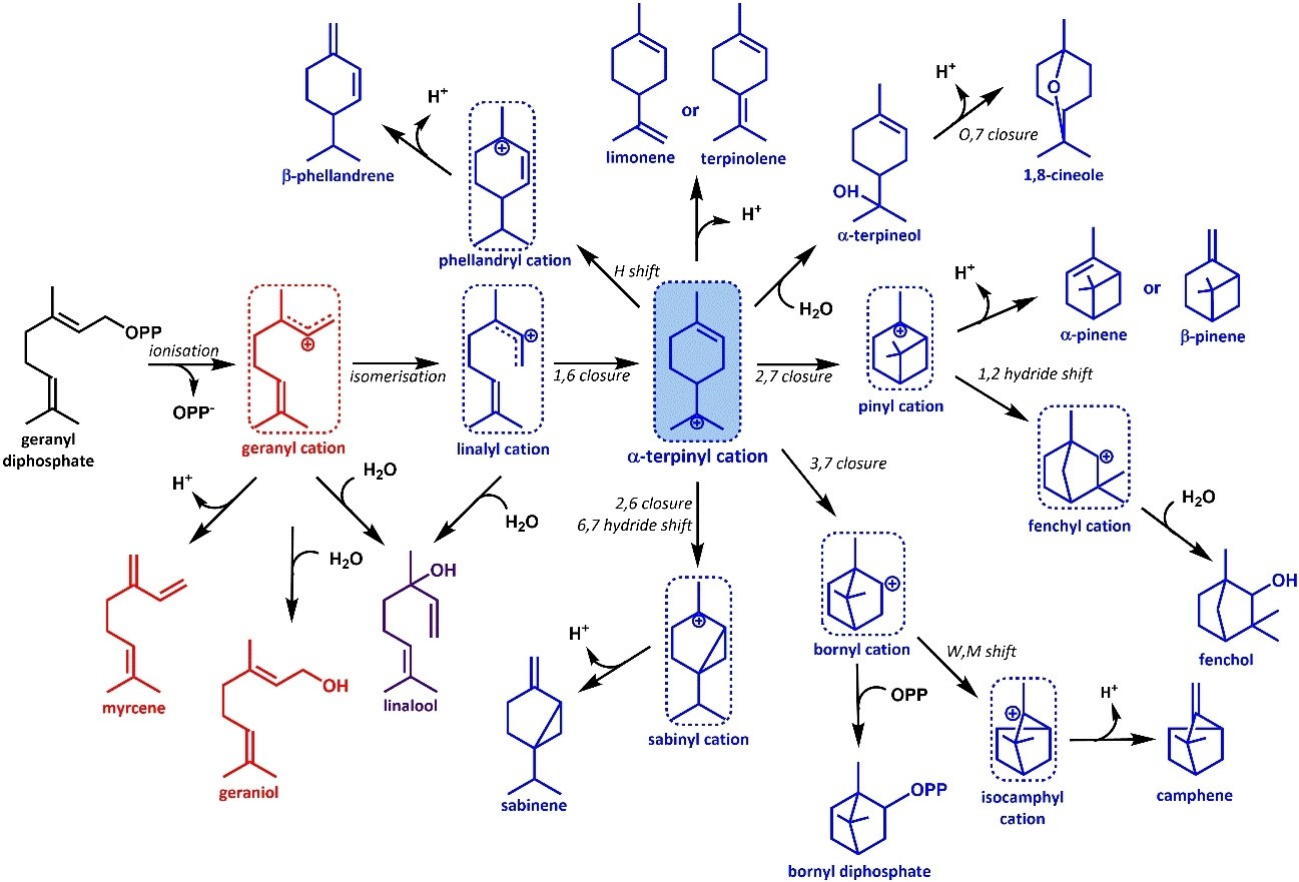
Branched monoterpene synthase catalysed reactions. All MTS catalysed reactions start with the ionisation of GPP resulting in the geranyl cation which can undergo a range of cyclisations and rearrangements before the reaction is terminated by deprotonation or nucleophilic attack. The formation of all cyclic monoterpenoid products requires the isomerisation of the geranyl cation to the linalyl cation via linalyl diphosphate (not shown), which can then cyclise to yield the α‐terpinyl cation. As both the (3*R*)‐linalyl and (3*S*)‐linalyl intermediates can be formed from the geranyl cation, divergent mirror image pathways exist in nature resulting in (+)‐ or (−)‐ products respectively.^[27]^ Products derived from the geranyl cation are shown in red, products derived from the linalyl cation are in blue, and products derived from both are in purple. Carbocation intermediates are shown in dashed boxes, and the α‐terpinyl cation is shaded in blue.

The highly branched reaction pathways of TSs is a major cause of the observed terpenoid product diversity. Two classes of TSs exist in nature, each using a different initiation reaction resulting in highly reactive carbocation intermediates. Class I TSs catalyse the metal‐dependent ionisation of the prenyl‐PP substrate, and class II TSs initiate the reaction via electrophilic activation of the substrate.^[19]^ The carbocation intermediates then react along one of several branches to form various linear or cyclic structures. As such chemical diversity is achieved because a single carbocation can undergo a range of cyclisations and hydride shifts before the reaction terminates via either de‐protonation or nucleophilic attack. Figure 1 shows proposed cyclisation cascades for monoterpene synthase catalysed reactions. In addition to the classical TSs mentioned above, a growing number of unusual TSs have been identified in recent years. These include the cyclopentane‐forming bi‐functional di/sesterterpene synthases that form a new type of class I TSs that is widely distributed in bacteria, fungi and plants,^[20]^ a group of chimeric TSs that possess both prenyltransfer and TS activities,^[21]^ as well as several non‐canonical TSs that catalyse TS‐like reactions but are not structurally related to class I or class II TSs.^[22]^ However, most known TSs belong to class I, which is the main focus of this review.^[23]^

Distantly related class I TSs from plants, fungi and bacteria all contain a strongly conserved class I TS fold containing the highly conserved metal‐binding motifs DDXXD and the NSE/DTE triad [(N,D)D(L,IV)X(S,T)XXE)],^[24]^ mutational analysis of these motifs confirmed their essential role in metal‐binding and catalysis.^[25]^ Unlike most other enzymes, the main catalytic challenge for TSs is not rate enhancement, but control of the highly reactive carbocation intermediates. As a result, after the initial ionisation, the TS active site does little else than to provide a suitable environment that facilitates reaction of the formed carbocations.^[26]^ There is a remarkable lack of sequence‐function relationship within the TS enzyme family, making product predictions from sequences alone virtually impossible. Here we review how recent advances in computational chemistry, high‐throughput (HTP) screening, and the use of data‐driven approaches have been used to explore more predictive engineering of TS activity. We focus on the origins of the multi‐reaction channel activity observed in class I TSs, with special emphasis on monoterpene synthases (MTSs) and sesquiterpene synthases (STSs).

## 2 Genome Mining for Terpene Synthase Activity

The broad spectrum of plant TS activity was first identified in crude plant extracts. TS encoding genes were subsequently isolated from plant material by exploiting the high levels of sequence similarity in amino acid sequences and gene organisation between TSs from the same or related plant species.^[28]^ Many soil bacteria, including *Streptomyces* species, have long been known for the production of the terpene derivatives 2‐methylisoborneol and geosmin, which are responsible for the characteristic earthy odours of moist soil,^[29]^ but only a few other terpenoids have been identified in extracts of bacterial culture, including *epi*‐cubenol^[30]^ and pentalenene.^[31]^

**Figure 2:**
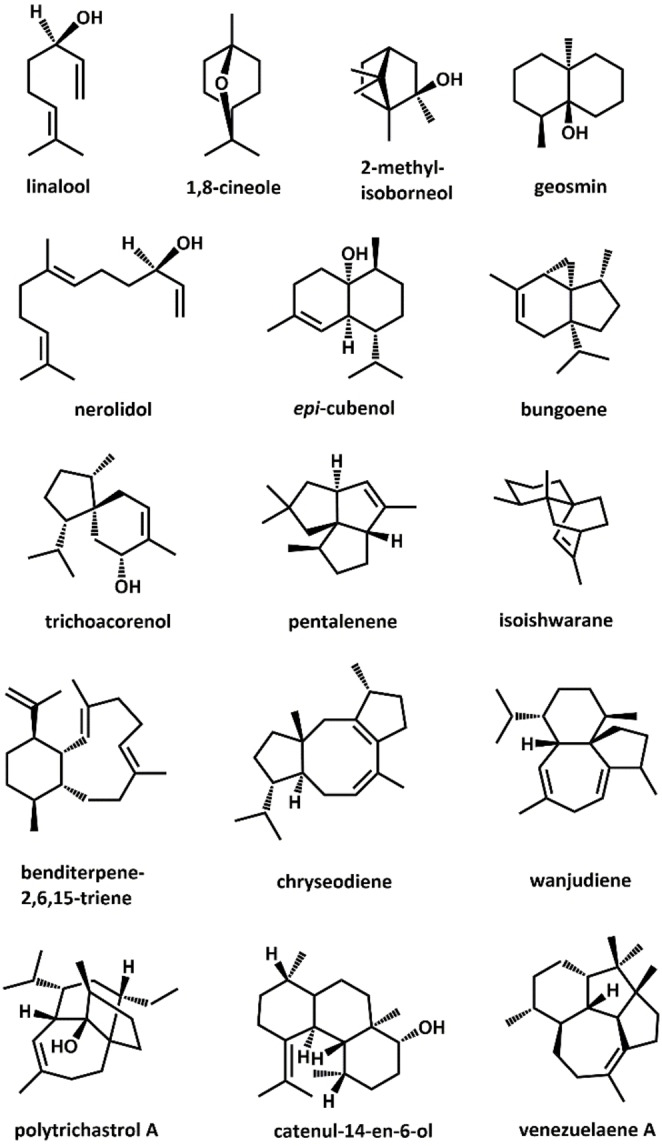
Structural diversity of terpenoids produced by bacterial terpene synthases. The products 2‐methyl‐isoborneol,^[29a]^ geosmin,^[29b]^ *epi*‐cubenol,^[30]^ and pentalenene^[31]^ have been discovered via classical isolation from bacterial culture extracts. Recent advances in genome mining techniques have allowed the identification of many new bacterial terpene synthases capable of producing a wealth of structurally diverse terpenoid skeletons, including the monoterpenoids linalool^[34b]^ and 1,8‐cineole,^[34a]^ the sesquiterpenoids nerolidol,^[34b]^ bungoene,^[35]^ trichoacorenol,^[36]^ and isoishwarane,^[37]^ and the diterpenoids benditerpene‐2,6,15‐triene,^[38]^ chryseodiene and wanjudiene,^[39a]^ polytrichastrol A,^[39b]^ catenul‐14‐en‐6‐ol,^[40]^ and venezuelaene A.^[41]^

With the wide availability of whole genome information, bacterial genomes have become a new source for mining TS activity. However, despite the overall conserved fold, the amino acid sequences of microbial TSs show no significant sequence similarities to related enzymes from plant origin. As such, conventional BLAST searches may not recognise sequences with overall low sequence similarity. Profile Hidden Markov Models containing the two signature metal binding motifs of class I TSs (PF03936) are effective in finding potential TSs in bacterial genomes, as they use statistical descriptions of consensus sequences for a given functional domain. This approach was first applied on bacterial genomes for the identification of 2‐methylisoborneol synthases in Actinomycetes.^[32]^ Since then, several similar approaches have identified hundreds of putative sesqui‐ and diterpene synthases in *Streptomyces* and other Actinomycetes.^[33]^ Using recombinant expression in heterologous hosts, the activities of a growing number of these enzymes have been established in recent years. A limited number of bacterial TSs with MTS activity have been identified, and include a 1,8‐cineole synthase (bCinS) and a bi‐functional linalool/nerolidol synthase (bLinS) from *S. clavuligerus*.^[34]^ However, genes encoding enzymes producing structurally diverse sesqui‐ or diterpenoids are more commonly found in bacterial genomes. Examples of recently identified bacterial STSs include a bungoene synthase from *S. bungoensis*,^[35]^ a trichoacorenol synthase from the actinobacteria *Amycolatopsis benzoatilytica*,^[36]^ and an isoishwarane synthase from *S. lincolnensis*.^[37]^ Examples of recently identified bacterial TSs producing diterpenoids include a benditerpene‐2,6,15‐triene synthase from *Streptomyces* sp. (CL12‐4),^[38]^ a chryseodiene synthase, a wanjudiene synthase, and a polytrichastrene synthase from the flavobacteria *Chryseobacterium polytrichastri* and *C. wanjuense*,^[39]^ a catenul‐14‐en‐6‐ol synthase from the actinomycete *Catenulispora acidiphila* ^[40]^ and a venezuelaene synthase from *S. venezuelae*, with the latter producing a unique 5‐5‐6‐7 tetracyclic skeleton.^[41]^ Figure 2 illustrates the structural diversity of terpenoids produced by bacterial TSs. In addition to the TSs mentioned above, a few bacterial class I TSs with sesterterpene synthase activity have also been identified, and include a multiproduct sestermobaraene synthase from *S. mobaraensis*,^[42]^ and a spata‐13,17‐diene synthase from *S. xinghaiensis* with promiscuous sesqui‐, di‐ and sesterterpene synthase activity.^[43]^ Promiscuous, multi‐substrate TS activity was first reported for a plant STS that also accepts GPP as a substrate, with each substrate yielding different cyclisation reactions.^[44]^ It has since been established that multi‐substrate TSs are widespread in plants.^[45]^ Substrate promiscuity also appears common in bacterial TSs, with bLinS as one of the earliest examples, exhibiting both linalool and nerolidol synthase activity.[^10a^, ^34b^, ^46^] A recent genome mining study identified over 2000 putative bacterial TSs in publicly available bacterial genome sequences from a wide range of bacterial species.^[47]^ Among the TSs tested for activity were several enzymes that catalysed promiscuous MTS and STS activity, including a nerolidol synthase and a bergamotene synthase from the radiation resistant bacterium *Rubrobacter radiotolerance*, a 10‐*epi*‐cubebol synthase and aromandendrene synthase from *Sorangium cellulosum*, as well as a previously identified geosmin synthase from *S. coelicolor* A3, suggesting that bacteria may be an unexplored rich source for further TS activity. The use of bacterial TSs over enzymes from plant sources is advantageous, as the former often express better in heterologous microbial hosts.

## 3 Structural Features Class I Terpene Synthases

**Figure 3:**
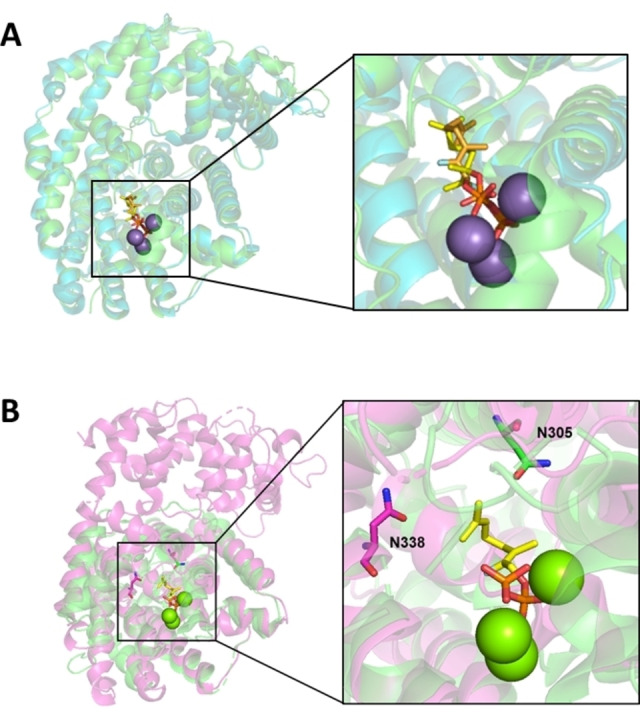
Structural features class I monoterpene synthases. A) Structural overlay of (*S*)‐(−)‐limonene synthase (green) from *Mentha spicata* (SLimS; PDB 2ONG)^[48]^ and (*R*)‐(+)‐limonene synthase (cyan) from *Citrus sinensis* (RLimS; PDB 5UV1).^[49]^ The Mn^2+^ ions are shown in purple spheres and the fluorinated substrate analogues are shown in yellow and orange sticks in SLimS and RLimS respectively. B) Structural overlay of bacterial 1,8‐cineole synthase from *S. clavuligerus* (bCinS; PDB 5NX7)^[46]^ (green) and plant 1,8‐cineole synthase from *Salvia fruticosa* (SfCinS; PDB 2J5C)^[50]^ (purple). The fluorinated substrate analogue and Mg^2+^ ions, as bound to bCinS, are shown in yellow sticks and green spheres, respectively. Asn305 in bCinS and Asn338 in SfCinS are indicated and shown as sticks. Panel B was reprinted without modifications from Ref. [51] with permission under the creative commons CC‐BY 4.0 licence.

The first crystal structures of TSs, the STSs pentalenene synthase from *Streptomyces* UC5319^[26a]^ and 5‐*epi*‐aristolochene synthase from tobacco (TEAS),^[24a]^ and the MTSs bornyl diphosphate synthase (BorS) from sage^[26b]^ and (*S*)‐(−)‐limonene synthase (SLimS) from spearmint,^[48]^ revealed a strongly conserved class I TS fold across distantly related TSs. Where most bacterial TSs consist of a class I TS domain only, most plant enzymes contain an additional N‐terminal domain of unknown function (Figure 3).

The class I TS fold consists of an α‐helical fold that forms the hydrophobic active site which is closed off from the bulk solvent by flexible loops located on the surface of the protein. Binding of the three metal ions and the pyrophosphate (PP) moiety of the substrate trigger a conformational change resulting in the closure of the active site offering protection from bulk solvent.^[26b]^ Comparison of the crystal structures of the open and closed (ligand‐bound) conformation of selinadiene synthase revealed an induced fit mechanism involving a structurally conserved effector triad comprising a PP sensor, linker and effector residue.^[52]^ A hydrophobic pocket is essential for TS chemistry as it avoids permanent enzyme inactivation via active site alkylation. In addition, the exclusion of water is required to avoid premature quenching of the reaction. Where carbocation reactions are normally facilitated in polar environments, TSs have evolved to stabilise the transient carbocation intermediates in a hydrophobic environment by carefully placed dipoles, such as backbone carbonyl groups, and aromatic residues, which can interact with the cations via their π system, without the need for negatively charged residues.^[53]^ In fact, a conserved structural feature of class I TSs is a helix break which orients a main‐chain carbonyl group into the active site, where the resulting negative electrostatic potential of the helix dipole stabilises carbocation intermediates during catalysis.^[54]^

Another mechanism employed by TSs to avoid by‐product formation is the creation of an active site contour that forces the substrate to bind in a product‐like conformation. This is observed for many TSs, and is particularly striking when the active sites of SLimS^[48]^ and (*R*)‐(+)‐limonene synthase^[55]^ are compared, where the enantiomeric selectivity of these enzymes is the result of the initial binding conformation of the GPP substrate (Figure 3, panel A). Similarly, the (*S*)‐(−)‐α‐terpineol intermediate is observed in bacterial cineole synthase (bCinS),[^51^, ^56^] where the (*R*)‐(+)‐α‐terpineol intermediate is accumulated in cineole synthase from sage (SfCinS),^[57]^ even though both reactions ultimately result in the achiral product 1,8‐cineole. Both water molecules involved in water attack of the α‐terpinyl cation leading to the formation of α‐terpineol are coordinated by Asn residues located at opposite ends of the active site (Figure 3, panel B).

## 4 Identification of Plasticity Regions and Targeted Engineering of Class I Terpene Synthases

Early domain swapping studies on chimeric enzymes confirmed TS activity is confined to the C‐terminal class I TS domain in plant TSs. These studies also revealed that a limited set of amino acids, located both in, and distant from, the active site, are responsible for product outcome.^[58]^ Similarly, site‐directed mutagenesis on highly homologous TSs that produce different products, allowed product channelling through alternative carbocation intermediates via only a few amino acid substitutions.[^27^, ^50^] However, in most cases altered product profiles were the result of premature quenching of the reaction due to a relaxed control over the carbocation intermediates and not stabilisation of alternative carbocation intermediates.[^25d^, ^59^]

**Figure 4:**
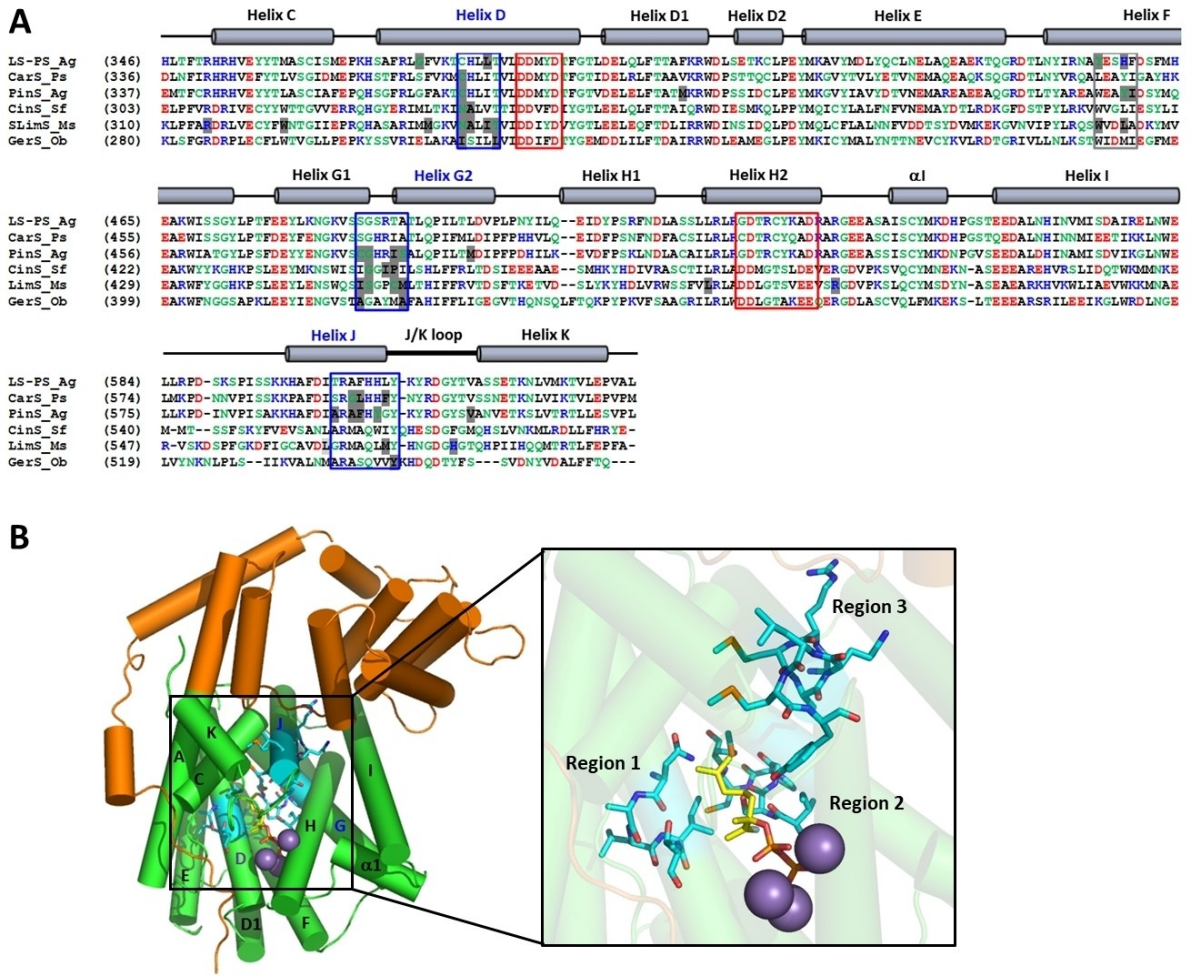
Identification and location of clustered plasticity residues in the plant MTS family. A) Multiple sequence alignment of the C‐terminal domains of MTS enzymes that have been mutated to variants with altered product profiles. Nonpolar residues are shown in black, polar residues in green, positively charged residues in blue, and negatively charged residues in red. The conserved DDXXD and NSE/DTE motifs are indicated with red boxes. Mutated amino acid residues that resulted in altered product profiles are highlighted in light grey. The three plasticity regions are marked with blue boxes. A potential fourth plasticity region is marked with a grey box. LS‐PS_Ag is (−)‐limonene/(−)‐α‐pinene synthase from *Abies grandis*,^[58a]^ CarS_Ps is (+)‐3‐carene synthase from *Picea sitchensis*,^[61]^ PinS_Ag is (−)‐pinene synthase from *Abies grandis*,^[27]^ CinS_Sf is 1,8‐cineole synthase from *Salvia fruticosa*,^[50]^ LimS_Ms is (−)‐(4*S*)limonene synthase from *Mentha spicata*,[^60^, ^62^] and GerS_Ob is geraniol synthase from *Ocimum basilicum*.^[63]^ The secondary structure of terpene synthases is indicated above the alignment.^[24a]^ B) The three identified plasticity regions mapped onto the structure of SLimS (PDB ID: 2ONG).^[48]^ The N‐terminal domain is shown in orange, the C‐terminal class I TS domain in green, the fluorinated substrate analogue in yellow, and the Mn^2+^ ions in purple. The three plasticity regions are highlighted in cyan. Reprinted with permission from Ref. [59]. Copyright 2018 American Chemical Society.

When several mutational studies on MTSs from plant origin are compared, patterns start to emerge. A residue just upstream of the DDXXD motif, although not conserved, was shown to be important in product outcome in several different enzymes. The presence of a polar residue (Asn345) at this position was shown to be essential for the formation of limonene in SLimS.^[60]^ Introduction of Ile to replace Asn338 in SfCinS resulted in sabinene as the main product, with the variant no longer able to support the water attack step.^[50]^ Similarly, Cys372 in pinene synthase (PinS) and Ser362 in camphene synthase from grand fir were implicated in channelling the carbocation intermediates down the correct path.^[27]^ A further comparison of mutational studies led to the identification of three structurally conserved functional plasticity regions in the family of plant MTSs, where residues involved in product outcome are clustered (Figure 4). Region 1 is located just upstream of the DDXXD metal‐binding motif, region 2 covers the helix break motif implicated in carbocation stabilisation, and region 3 is located near the C‐terminus. Introduction of consensus sequences for these plasticity regions in different plant MTSs with increasingly complex cyclisation cascades, suggested that the first two plasticity regions are involved in the formation and stabilisation of cations early in the cyclisation cascade, where mutations have drastic effects on the product outcome. The third region is implicated in fine‐tuning product profiles in the latter stages of the cyclisation cascade of enzymes that catalyse more complex cyclisation cascades resulting in bi‐cyclic products.^[59]^

Recent mutational studies on several bacterial TSs yielded valuable insights into how subtle remoulding of the active site contour, often involving aromatic residues, can lead to the accumulation of alternative products. The substitution of aromatic residues with polar residues in the active site of the STS *epi*‐isozizaene synthase resulted in variants that exhibited an expanded product chemo‐diversity.^[64]^ Phe76, positioned in aforementioned region 1 in pentalenene synthase is involved in stabilisation of a positive charge which supports subsequent cyclisation involving less stable secondary carbocations rather than the much more stable tertiary carbocations stabilised in most other STSs. An aromatic residue at this position has been implicated in a similar role in other bacterial STSs involving secondary carbocations.^[65]^ The I66F mutation in the diterpene synthase polytrichastrene synthase resulted in a major functional shift and the accumulation of several new products not produced by the native enzyme.^[39b]^

More systematic mutagenesis approaches yielded insights into the mutability landscape of TSs. The reciprocal inter‐conversion of catalytic specificity between two distinct, but evolutionary related STSs, TEAS and premnaspirodiene synthase, allowed the mapping of structural features responsible for functional attributes. It was shown that catalytic specificity indeed relies on active site contour, but crucially, the importance of supporting layers of residues that surround the active site was exposed. The latter may affect catalysis via altering the position or dynamic properties of the active site residues relative to the carbocation intermediates.^[66]^ A follow up study on the same enzymes involved the quantitative exploration of the catalytic landscape of these divergent plant STSs, and revealed a rugged landscape in which alternative catalytic specificities are accessible via relatively few mutations.^[67]^

These systematic mutagenesis studies demonstrate the underlying evolvability of the class I TS fold. Interestingly, the identified plasticity residues generally play a shared role in the catalytic outcome, i. e. the mutations introduced were effectively additive, increasing the likelihood that rational design ideas can be applied to class I TS provided that enough information about the fitness landscape is available

## 5 Computational Chemistry of Terpene Synthases

The use of computational chemistry methods can provide valuable insights in TS catalysed reactions that are not accessible experimentally. Quantum mechanical (QM) calculations are used to study gas phase carbocation chemistry, modelling and bound states are studied using molecular dynamics (MD), and in‐enzyme reactions are studied using multiscale methods such as quantum mechanics/molecular mechanics (QM/MM), and account for the effects of enzyme environment, cofactors, solvent and salt.^[68]^

Computational prediction of the correct fold, and therefore shape of the active site in the absence of a crystal structure is challenging, although this is enabled by recent advances in structure predictions such as AlphaFold.^[69]^ However, even if structural information is available, the prediction of carbocation binding modes is not straightforward, as the carbocation inter‐mediates in TSs are only weakly bound with no hydrogen bonds or ionic interactions. TerDockin, which comprises a series of computational protocols, was developed to predict the binding orientation, including stereo‐chemical preference, of the olefin moiety of prenyl‐PP substrates and their derived carbocations in relation to PP bound in the active site of TSs.^[70]^ Similarly, EnzyDock is a multiscale consensus docking program that can predict chemically relevant orientations and conformations for substrate, intermediates and product, and has been successfully applied to TSs.^[71]^ A combination of QM calculations and computational docking was used to generate an all‐atom model of all putative intermediates in the reaction catalysed by TEAS, resulting in a high‐resolution model of the reaction inter‐mediates.^[72]^ Several docked poses along the same reaction coordinate were achieved for BorS, and the effect of a water molecule in the active site proved to be significant. In‐depth knowledge of binding modes of key intermediates, and the effect of active site water molecules provides valuable information for rational design of TSs that is lacking from crystal structures alone. A computational work‐flow, consisting of homology modelling, substrate docking and enumerating possible carbocation inter‐mediates from the farnesyl cation in the active site, has been developed for the prediction of hydrocarbon product skeletons, and was successfully applied to predict a linear triquinane product skeleton for a putative bacterial STS sequence,^[73]^ providing the first example of product prediction from a protein sequence.

Computational studies have revealed crucial insights into the strategies used by TSs to control reactive carbocation inter‐mediates. QM/MM free‐energy simulations have shown that TSs gain chemical control over the reactive carbocation intermediates by raising the energy barriers of branch‐point intermediates to by‐pass the formation of unwanted side‐products. This is in contrast to most other enzymes that reduce the free‐energy barriers of the transition state to achieve enhanced reaction rates.^[74]^ Tuning of these energy barriers is achieved by modulation of electrostatic interactions in the unique binary active site of TSs.^[7]^ In the charged region, covering the metal ion and PP binding sites, the PP moiety itself has the largest influence on the chemistry where it slows down the intrinsic carbocation reactivity early on in the reaction cascade, thereby avoiding early side reactions.^[75]^ Many mutagenesis studies focussed on the hydrophobic region of the active site, where aromatic residues such as Phe have a more obvious and direct role in guiding the reactive intermediates via steric or cation‐π interactions. However, these computational studies show that modulating the electrostatic interactions via mutations in the charged region of the active site may have a big impact on product outcome.

Ever since the first crystal structures became available for TSs the important role of bulky aromatic residues in active site contouring and carbocation stabilisation has been recognised. However, the number of aromatic residues in the active site of different TSs varies. bCinS contains four Phe residues restricting the active site volume of the enzyme, which provides a rationale for the fact it does not accept FPP as a substrate unlike the related enzyme bLinS.^[46]^ Additionally, a larger number of aromatic residues has been implicated in enzyme fidelity in aristolochene synthase from *Aspergillus terreus*. QM/MM MD simulations revealed that the carbocations are guided at each step via strict conformational restraints or cation‐π interactions from additional aromatic residues that are not present in TEAS, the latter being a much more promiscuous enzyme. The absence of cation‐π interactions in TEAS resulted in higher reaction barriers and less heat release in the latter stages of the cyclisation cascade.^[76]^ In line with this, the aromatic rich active site of bCinS is also capable of catalysing a high fidelity cyclisation cascade resulting in >95 % pure bi‐cyclic cineole formation. More research is required to assess if the number of bulky aromatic residues in the active sites of TSs could serve as a predictor of product fidelity.

**Figure 5:**
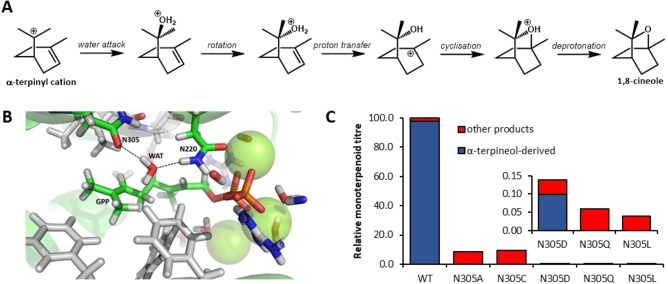
Asn305 controls water attack in bCinS. A) Proposed mechanism for cineole formation by bCinS from α‐terpinyl.^[46]^ B) The active site of wild‐type bCinS showing GPP (green carbon atoms) and a water molecule coordinated by N305 and N220 in a representative structure from cluster analysis of the MD trajectory. C) Relative product profiles obtained for wt‐bCinS and the bCinS‐N305 variants. Total products derived from α‐terpineol are in blue and other products are shown in red. Panels B and C were reprinted from Ref. [51] with panel C plotted in a modified way, with permission according to creative commons license CC BY 4.0.

Computational chemistry has also been applied to study the quenching mechanisms of TS catalysed reactions. Deprotonation is the most common termination mechanism, but the catalytic base for proton abstraction is not always obvious. Computational approaches supplied further evidence for the involvement of PP,^[76]^ the employment of non‐traditional bases such as Ser,^[77]^ as well as the participation of multiple bases in the final deprotonation step.^[59]^ The use of multiple acid/base pairs has been implicated in increased promiscuity in TSs, offering a multitude of termination options.^[76]^ The nucleophilic water attack mechanism of bCinS has been studied extensively both experimentally and computationally. A key step in the bCinS cyclisation cascade is water attack on C7 of the α‐terpinyl cation resulting in the α‐terpineol intermediate (Figure 5, panel A). In the bCinS crystal structure in complex with a fluorinated GPP analogue, the otherwise hydrophobic active site, contains a single water molecule close to C7 of the substrate analogue, which is coordinated by Asn220 and Asn305[^46^, ^51^] (Figure 5, panel B). MD simulations on bCinS show that this water molecule remains at an average distance of 3.8 Å to C7 of GPP.^[46]^ Mutational analysis of Asn305 resulted in variants that were no longer able to efficiently form α‐terpineol, producing mostly non‐hydroxylated products re‐directed from the α‐terpinyl cation (Figure 5, panel C).^[51]^ The crucial role of Asn305 in water‐coordination was confirmed by further MD simulations, revealing a disrupted water network in all variants. bCinS is unusual, as most TSs do not produce hydroxylated products and avoid water in the active site. For those that do catalyse a water capture step, clearly defined water molecules and active site residues involved are often lacking. A recent study on the STS germacradien‐11‐ol synthase revealed that subtle changes in the water binding regions of TSs can have profound effects on the hydroxylation activities exhibited by these enzymes.^[78]^

## 6 Sequence Diversity and High‐Throughput Screening for Terpene Synthase Activity

So far the methods discussed largely gained insights into the reaction mechanism of individual enzymes. Data‐driven approaches such as machine‐learning (ML) can produce models that give additional insights into the reaction chemistry of the class I TS enzyme family as a whole. However, ML algorithms are highly “data‐hungry” often requiring a large number of sequence‐function observations to reach acceptable performance levels. This sequence‐function data can be obtained from natural occurring sequence diversity or generated artificially via directed evolution (DE), the latter being heavily reliant on the availability of efficient high‐throughput (HTP) screening assays.

**Figure 6:**
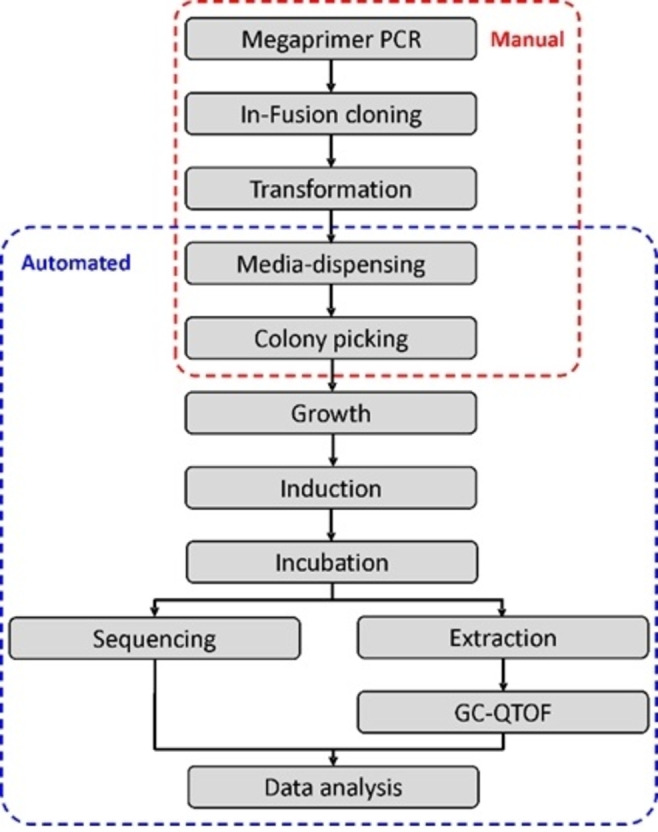
Schematic overview of the automated pipeline for diverse monoterpenoids detection. The pipeline is designed for use of 96‐well plates to ensure compatibility with robotic liquid handling platforms for easy sample processing. Manual and automated steps are indicated with red and blue dashed boxes respectively. This figure was reprinted without modifications from Ref. [83]. with permission according to creative commons license CC BY 4.0.

The development of suitable HTP screening assays for TS activity is particularly challenging due to the fact that multiple volatile products may be produced from a single substrate. Several screening assays for the detection of enhanced TS activity have been developed, such as colorimetric detection of PP by‐product release using malachite green,^[79]^ or colorimetric monoterpenoid detection using hydrophobic dyes.^[80]^ Other assays allow the detection of enhanced terpene production *in vivo* in engineered microbes, where terpene formation competes with other artificial pathways for a shared isoprenoid substrate pool,^[81]^ or include genetically encoded biosensors that detect intracellular isoprene concentrations.^[82]^ However, none of these methods allow rapid detection of multiple volatile terpenoid compounds, which is crucial to obtain large TS sequence‐function datasets. A recently reported automated GC‐MS pipeline allows the detection of diverse monoterpenoids *in vivo* in engineered *Escherichia coli*.^[83]^ The pipeline was designed for use with 96‐well plates to ensure compatibility with robotic liquid handling platforms, as well as analysis using a GC‐MS equipped with a 96‐well plate auto‐sampler. Rapid data analysis was achieved using automated data extraction scripts (Figure 6). The pipeline was used to screen PinS variant libraries with mutations in the aforementioned plasticity regions, resulting in over 70 variants with altered product profiles. Additionally, the results gave insight into the amino acid positions with the greatest contribution to functional plasticity in PinS and the significance of the helix break motif in carbocation cyclisation. Even though automation has improved the throughput of this screening assay significantly, the reliance on GC‐MS for the detection of volatile products remains a bottleneck in rapid terpenoid screening to date. The availability of dyes and biosensors specific for certain terpenoids or groups of terpenoids, such as the transcription factor CamR, developed as biosensor for bicyclic monoterpenes described in a recent preprint,^[84]^ could pave the way for ultra‐HTP screening of complex terpenoid mixtures in the near future.

## 7 Data‐Driven Approaches for Functional Prediction of Terpene Synthase Activity

As mentioned previously, TS sequences are highly diverse, and sequence similarity amongst plant TSs is strongly influenced by phylogeny,^[85]^ this and the lack of clear sequence‐function relationships amongst TSs demands the use of more sophisticated computational approaches for functional prediction. ML methods can find patterns in multi‐dimensional datasets and generate functional models from these data. Various ML algorithms with applications in protein engineering have been developed, and range from simple linear models to highly complex neural networks. In depth discussion of these methods is outside the scope of this review, and excellent recent reviews on this topic already exist.^[86]^

An early example of the application of computer algorithms to predict combinations of mutations in a TS was performed on γ‐humulene synthase, a promiscuous STS generating >50 products. The enzyme was first subjected to active‐site saturation mutagenesis to identify plasticity residues, which were then systematically recombined using a mathematical model that predicts a set of mutations based on how much the product distribution moved towards the desired product for a given combination of mutations. This approach yielded several variant synthases with narrower product specificities than the native enzyme.^[87]^ These results are in contrast to previously mentioned site‐directed mutagenesis studies where altered products were often the result of premature quenching leading to more diverse product profiles.

**Figure 7:**
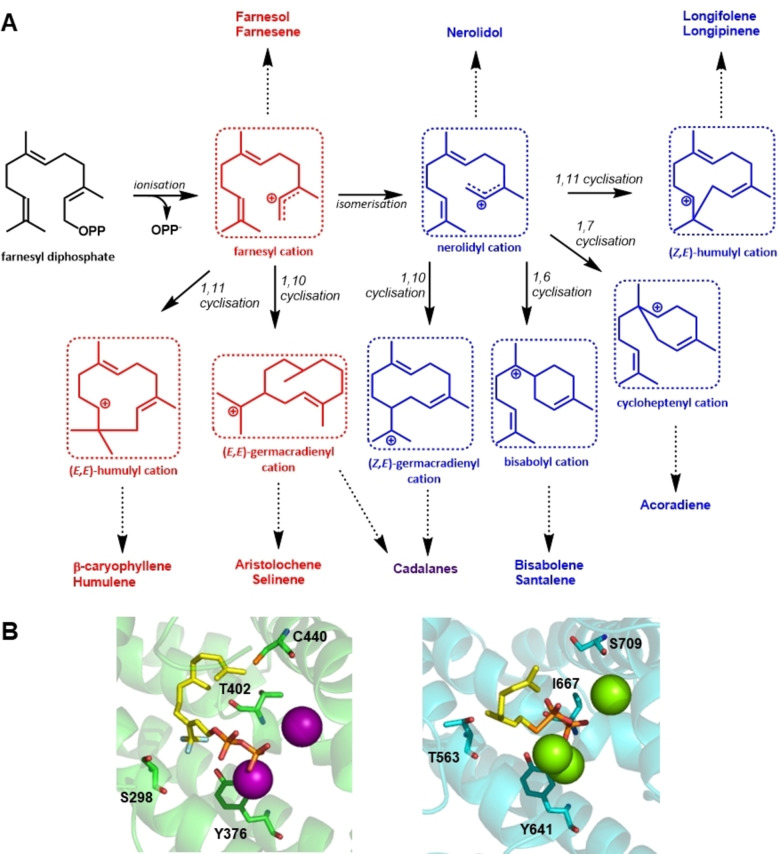
Identification of cation‐specific co‐evolved residues in plant sesquiterpene synthases.^[88]^ A) Key carbocation intermediates in STS catalysed reactions. All STS catalysed reactions start with the ionisation of FPP resulting in the farnesyl cation. The farnesyl cation is converted to the nerolidyl cation following isomerisation. Both the geranyl and nerolidyl cations can produce linear products or various cyclic intermediates. Products derived from the farnesyl cation are shown in red, products derived from the nerolidyl cation are in blue, and products derived from both are in purple. B) Cation specific co‐evolved residue pairs in a farnesyl cation specific enzyme (TEAS, PDB 5EAU; green, left)^[24a]^ and a nerolidyl specific enzyme (BIS, PDB 3SDU; cyan, right).^[89]^ Co‐evolved residue pairs (S298‐T402 and Y376‐C440 in TEAS, and T563‐I667 and Y641‐S706 in BIS) are indicated and shown in sticks, fluorinated substrate analogues are shown in yellow sticks, and Mg^2+^ ions are shown as spheres.

A recent study combined different computational approaches, including homology modelling, ML and co‐evolutionary analysis, to predict precursor cation specificity for plant STSs.^[88]^ Multi‐product STSs show a high degree of selectivity towards either the farnesyl or nerolidyl cation, which is determined early on in the cyclisation cascade. Analogous to MTS catalysed reactions, the FPP substrate undergoes metal‐dependent ionisation resulting in the farnesyl cation, which can undergo isomerisation to the nerolidyl cation. However, in contrast to MTS catalysed reactions where the isomerisation from geranyl to linalyl is an essential step towards cyclic products via α‐terpinyl, both farnesyl and nerolidyl can undergo various cyclisation reactions resulting in a multitude of possible products (Figure 7, panel A). A ML approach, combining both sequence and structural information, was applied to accurately predict cation specificity for plant STSs. The inclusion of structure‐derived information and homology modelling avoids the phylogeny bias that would arise if sequence information alone was used. The identified cation‐specific residues were located in five structural regions which, for a large part, overlap with the plasticity regions previously identified in plant MTSs.^[59]^ A subsequent correlated mutation analysis on the identified cation‐specific residues on over 8000 uncharacterised plant STS sequences resulted in the identification of several cation‐specific co‐evolved residue pairs. One such pair is Thr402 and Ser298 in TEAS, a farnesyl specific enzyme, where the dipole of Thr402 has been implicated in directing the cationic end of the farnesyl chain into the active site in preparation for 1–10 cyclisation.^[24a]^ The equivalent pair in α‐bisabolene synthase (BIS) consists of Ile667 and Thr563, where the inert Ile is not capable of performing this function in nerolidyl specific enzymes. A second residue pair was identified involving Tyr376 and Cys440 in TEAS and Tyr641 and Ser709 in BIS, (Figure 7, panel B) the latter pair has been implicated in the formation of the nerolidyl cation and subsequent further cyclisation in nerolidyl specific STS.^[88]^ Interestingly, the co‐evolved residue pairs identified in plant STSs are conserved in plant MTSs, and include residues in the previously identified plasticity regions 1 and 2, which are known to have an effect on the early stages of the cyclisation cascade.[^59^, ^83^] Further research is required to establish if these models can be applied as a predictor for linear vs. cyclic products in MTSs.

## 8 Summary and Outlook

TSs are intriguing enzymes that catalyse the high‐energy cyclisation of reactive carbocations in highly branched cyclisation cascades. These reactions often result in the formation of multiple terpenoid products which hampers the use of TSs in biotechnological applications for the production of industrially desirable terpenoid products. To prevent enzyme inactivation, TSs have evolved to ‘manage’ the reactive carbocations in relatively inert, hydrophobic active sites. This lack of direct interaction with the reaction intermediates has resulted in an overall lack of sequence‐function correlation amongst TSs, and product predictions from the amino acid sequence alone remains elusive to date.

Despite their overall low sequence similarity, through the use of clever data‐mining tools and the growing availability of genome sequence data the discovery of novel TS activities has accelerated in recent years. Structural analysis revealed a highly conserved fold for all class I TSs. The availability of structural and mutagenesis data has resulted in the identification of conserved areas of plasticity collectively responsible for product outcome. The critical involvement of aromatic residues in guiding the reactive intermediates via placing steric constraints and cation‐π interactions is a common feature in TSs. In fact, the number of aromatic residues in the active site may be used to predict if an enzyme is likely to be promiscuous or not, with high product fidelity seemingly being correlated with a larger number of aromatic residues. Furthermore, crystallographic studies have shown that substrates bind in product‐like conformations. Computational docking tools have been developed that allow the prediction of substrate and carbocation binding modes, including stereo‐chemical preferences, paving the way for the prediction of product properties from protein sequences, provided that tools such as the recently developed AlphaFold for the highly accurate prediction of protein structures, allow precise prediction of the active site contour.^[69]^

The continuing development of HTP assays for the detection of diverse volatile terpenoids, will allow the screening of larger TS variant libraries increasing the chance of finding TSs with desired properties in DE experiments. In addition, these experiments will lead to large sequence‐function data‐sets which, along with the wealth of putative TS sequences stored in public databases can be used in data‐driven ML approaches, ultimately leading to designer TS activities. It is important to note that HTP screening can only detect known compounds, so classical isolation experiments will also continue to play an important role in the discovery of new TS activities.

**Figure 8:**
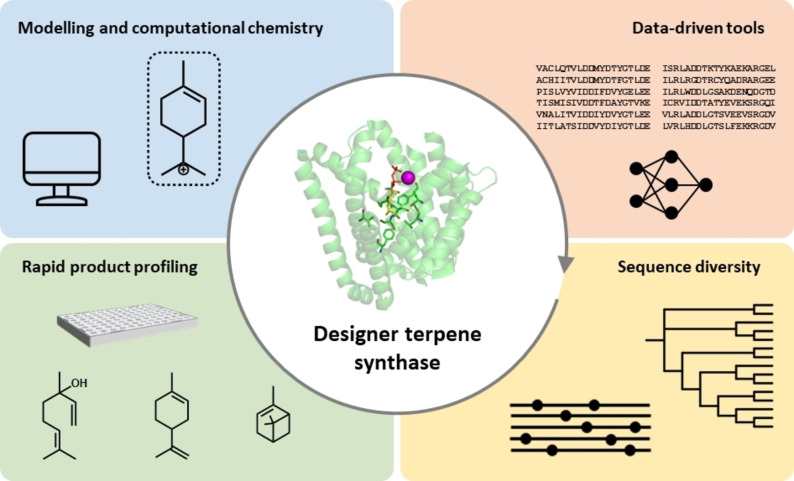
Multidisciplinary approach towards predictive engineering of terpene synthases. Engineering of designer TS activity is enabled via integration of multidisciplinary approaches, including generation of sequence diversity, genome mining, HTP product profiling aided by automation, modelling and computational chemistry, and data‐driven computational tools.

In conclusion, through recent advances in the availability of genome information and mining tools, efficient DE protocols and the development of HTP screening technologies, including laboratory automation, as well as sophisticated computational tools, it is increasingly possible to acquire a holistic picture of the TS enzyme family and TS chemistry. And by combining the wealth of available information the prediction of function from sequence is becoming a reality (Figure 8).

## Conflict of interest

The authors declare no conflict of interest.

## Biographical Information

*Nicole Gerharda Henrica Leferink received her BSc in Biochemistry and Biotechnology from the Saxion University of Applied Sciences and her MSc and PhD in Biochemistry from Wageningen University. Prior to joining the Future Biomanufacturing Research Hub as Research Fellow, she conducted postdoctoral research in molecular enzymology at the Manchester Institute of Biotechnology. Her main research interests are rational engineering and directed evolution towards the development of designer enzymes for applications in biomanufacturing*.

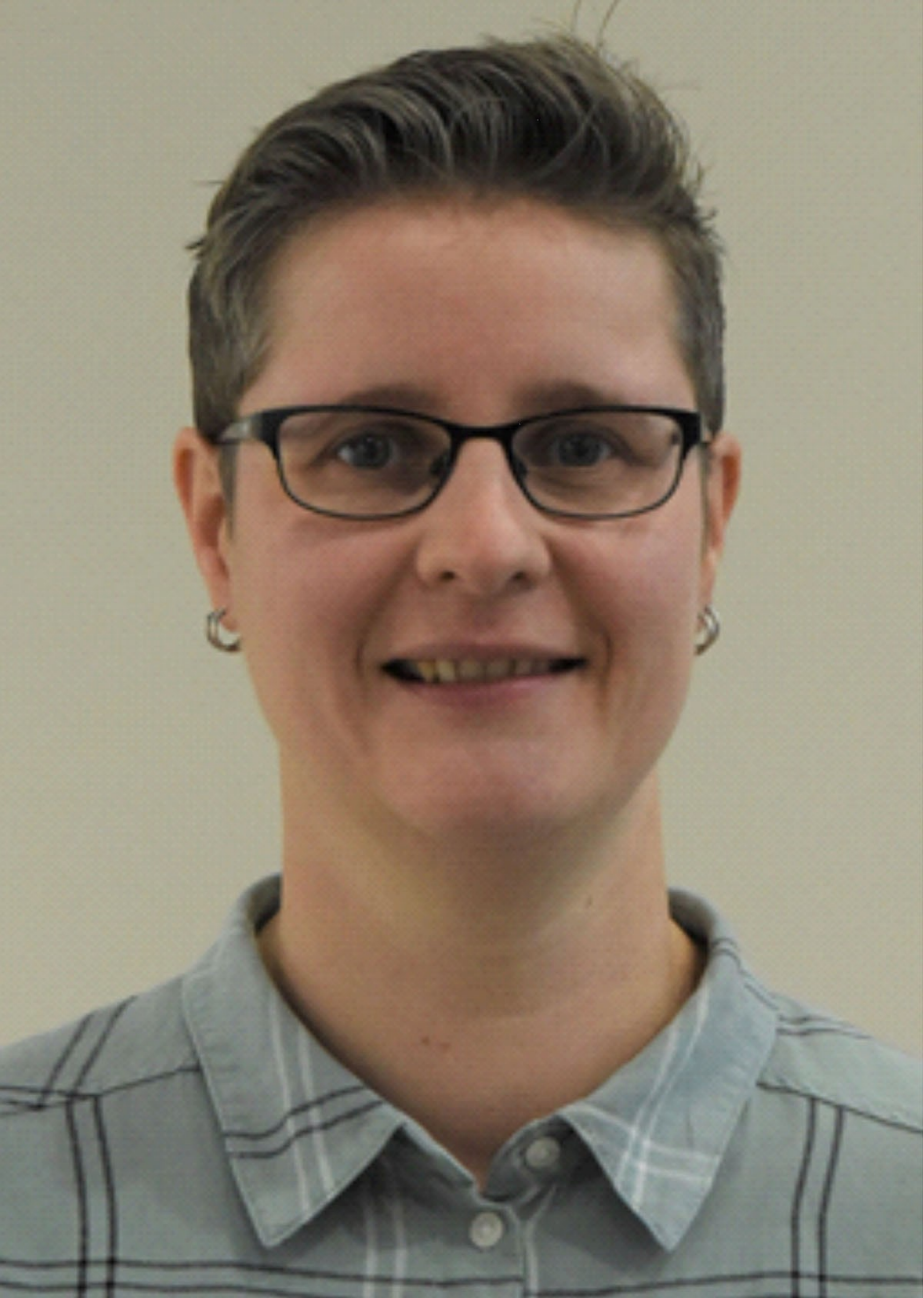

## Biographical Information

*Nigel Shaun Scrutton is Professor of Molecular Enzymology and Biophysical Chemistry at the University of Manchester. He received his BSc from King's College, University of London, and his PhD and ScD degrees from the University of Cambridge. His research interests are focused on enzyme catalysis from structural, mechanistic and kinetic perspectives, synthetic biology and the biomanufacturing of chemicals, materials and synthetic fuels. He is Director of the UK Future Biomanufacturing Research Hub and the Synthetic Biology Research Centre SYNBIOCHEM. He co‐founded the fuels from biology company C3 BIOTECH in 2015*.